# Therapieresistenz oder Non-Compliance? Zusammenhang zwischen einer gastrointestinalen Resorptionsstörung und einer Therapieresistenz bei Depression

**DOI:** 10.1007/s00115-023-01585-2

**Published:** 2023-12-12

**Authors:** S. Wudy, R. Regenthal, G. Schomerus, M. Strauß

**Affiliations:** 1 Klinik und Poliklinik für Psychiatrie und Psychotherapie, Universitätsklinikum Leipzig, AöR, Semmelweisstr. 10, 04103 Leipzig, Deutschland; 2 Klinische Pharmakologie, Rudolf-Boehm-Institut für Pharmakologie und Toxikologie, Universität Leipzig, Leipzig, Deutschland

## Hintergrund

Depressionen gehören zu den häufigsten psychischen Störungen. Die Wahrscheinlichkeit, an einer Depression zu erkranken, liegt weltweit bei 16–20 %. Deutschlandweit erkranken insgesamt 3 Mio. in einem Zeitraum von 12 Monaten [5].

Unipolare Depressionen sind gekennzeichnet durch eine grundlose depressive Stimmung, Freud- sowie Interessenlosigkeit, Antriebshemmung, verminderter Konzentration und Aufmerksamkeit, Schuldgefühlen aber auch Suizidgedanken oder -handlungen [4, 6]. Die Behandlung erfolgt in der Regel multimodal, idealerweise durch eine Kombination von medikamentösen und psychotherapeutischen Therapieverfahren. Aber auch eine Reihe nichtmedikamentöser Verfahren, wie z. B. nichtinvasiven Stimulationsverfahren, stehen als Behandlungsoptionen zur Verfügung [2].

In Deutschland sind zahlreiche antidepressive Medikamente zugelassen, welche meistens zu einer intrasynaptischen Erhöhung von Serotonin und/oder Noradrenalin führen [2].

Trotz der Verfügbarkeit wirksamer Therapiemethoden wird angenommen, dass 20–40 % der Betroffenen nur unzureichend oder gar nicht auf eine antidepressive Behandlung ansprechen [8]. In diesen Fällen spricht man von einer schwer behandelbaren oder therapieresistenten Depression (TRD) [1]. Eine einheitliche Definition einer TRD existiert nicht. In der nationalen Versorgungsleitlinie wird Therapieresistenz als das Nichtansprechen auf eine initiale Therapie sowie mindestens einer weiteren Behandlungsstrategie definiert [2].

Therapieresistente Depressionen sind nach wie vor eine klinische Herausforderung. Eine wichtige mögliche Ursache einer Therapieresistenz kann neben organischen Ursachen auch eine fehlende Compliance der Patient:innen sein. Vor diesem Hintergrund möchten wir an einem konkreten Fallbeispiel das Vorgehen bei einer TRD aufzeigen und mögliche Fallstricke diskutieren [2].

## Kasuistik

Berichtet wird über einen 54-jährigen Patienten mit einer schweren depressiven Episode, welcher aufgrund einer bisherigen Therapieresistenz auf unserer Spezialstation für Affektive Störungen aufgenommen wurde. Zum Aufnahmezeitpunkt zeigte sich der Patient affektiv starr, im Kontakt teilweise mutistisch und antriebsgehemmt. Auffällig war, dass er ungefragt beteuerte „seine Medikamente wie verordnet einzunehmen“. Die Behandlung der aktuellen depressiven Episode begann bereits vor 3 Jahren stationär. Mit einer Kombinationsbehandlung aus Sertralin und Olanzapin konnte zum damaligen Zeitpunkt eine Teilremission erreicht werden. Die Weiterbehandlung wurde durch einen niedergelassenen Psychiater fortgeführt, welcher nichts an der Entlassungsmedikation änderte. Leider verübte der Patient 4 Wochen nach der Entlassung einen schweren Suizidversuch in der Häuslichkeit, bei dem er sich 3 tiefe abdominelle Stichverletzungen zufügte. Nach der operativen Versorgung der Verletzungen wurde der Patient erneut in eine psychiatrische Klinik verlegt, da die depressive Symptomatik exazerbiert war. Ab diesem Zeitpunkt erfolgten mit kurzen Unterbrechungen insgesamt 6 stationäre sowie eine teilstationäre psychiatrische Behandlung. Ganz anders als bei der allerersten stationären Behandlung konnte trotz mehrerer antidepressiver Mono- und Kombinationstherapien (u. a. mit Sertralin, Bupropion, Venlafaxin, Clomipramin) als auch Augmentationsstrategien mit Lithiumcarbonat und mehreren atypischen Antipsychotika (u. a. Aripiprazol) keinerlei Therapieansprechen mehr auf die medikamentöse antidepressive Behandlung erreicht werden. Eine umfangreiche Diagnostik inkl. Labor- und EKG-Untersuchungen, toxikologischem Screening, Liquordiagnostik, cCT sowie cMRT ergaben keine Hinweise für eine organische Ursache für die Therapieresistenz. Ein therapeutisches Drug-Monitoring (TDM) erfolgte erstmalig während des 6. stationären Aufenthaltes. Der Befund eines negativen Plasmaspiegels für die zu diesem Zeitpunkt eingenommene Medikation (Lithiumcarbonat, Mirtazapin, Milnacipran und Clozapin) wurde als Non-Compliance des Patienten interpretiert.

Nach Aufnahme in unsere Klinik behandelten wir mit 100 mg Sertralin, 1200 mg Lithiumcarbonat und 1 mg Risperidon. Das TDM nach 7 Tagen Behandlung führte ebenfalls zu negativen Ergebnissen. Eine Non-Compliance konnte als Ursache ausgeschlossen werden, da die Einnahme der Medikation unter Aufsicht geschah. Die Kolleg:innen aus der klinischen Pharmakologie hielten einen pharmakogenetisch bedingten Phänotyp eines gesteigerten Metabolismus im Sinne eines „rapid metabolizers“ oder „ultra-rapid metabolizers“ des hepatischen Zytochrom 2D6 und eine Metabolisierungsstörung des Zytochrom 3A4 für unplausibel, da für alle Substanzen nach 1 Woche keinerlei Metaboliten im Plasma nachweisbar waren. Allerdings wurde aufgrund der Historie einer abdominellen Operation mit Entfernung von 26 cm Ileum eine gastrointestinale Resorptionsstörung diskutiert. Demzufolge wechselten wir die Applikationsform der antidepressiven Medikation von oral auf parenteral und verabreichten Citalopram i.v. Das TDM ergab daraufhin suffiziente Medikamentenspiegel (Tab. 1). Zwischenzeitlich erfolgte als Plausibilitätsprüfung kurzzeitig erneut ein Wechsel der Applikationsform von i.v. auf oral, was wieder zu negativen Ergebnissen im TDM führte. Unter der erneut angesetzten i.v. Therapie mit Citalopram war im Verlauf ein Ansprechen zu verzeichnen, allerdings nicht in ausreichender Form. Aufgrund fehlender alternativer medikamentöser Augmentationsmöglichkeiten kombinierten wir mit einer Elektrokonvulsionstherapie.

**Table 1:** 

	Citalopram (ug/l) (Referenzbereich 50–110)	Desmethylcitalopram (ug/l)
*Tag 1 – Citalopram 20* *mg i.v.*		
Spitzenspiegel 1 h	15,7	< 2,0
Spitzenspiegel 3 h	11,5	< 2,0
*Tag 3 – Citalopram 40* *mg i.v.*		
Spitzenspiegel 1 h	42,7	3,8
Spitzenspiegel 3 h	52,3	4,1
*Tag 14 – Citalopram 40* *mg p.o.*		
Spitzenspiegel 1 h	14,3	8,6
*Tag 35 – Citalopram 40* *mg i.v.*		
Spitzenspiegel 1 h	56,2	17,3
*Tag 47 – Citalopram 40* *mg i.v.*		
Talspiegel	91,8	30,2

Unter dieser Therapie kam es zu einer deutlichen Besserung der Symptomatik, sodass die Entlassung nach Hause erfolgen konnte. Als Erhaltungstherapie wurde die i.v. Gabe von Citalopram durch den Hausarzt organisiert. Eine Gabe von 3‑mal wöchentlich wurde anhand der pharmakokinetischen Eigenschaften des Präparates als ausreichend eingeschätzt.

## Diskussion

Die Gründe für ein negatives TDM können sehr vielfältig sein und schließen unter anderem Medikamenteninteraktionen, interindividuelle pharmakokinetische Unterschiede, aber auch eine mangelnde Compliance ein. Auch sollte an seltene Ursachen wie eine gastrointestinale Resorptionsstörung gedacht werden, wie es bei unserem Patienten der Fall war [2].

Die Literatur bezüglich des Einflusses gastrointestinaler Operationen und dadurch bedingter Resorptionsstörungen auf die Pharmakokinetik antidepressiver Medikamente ist spärlich. Allerdings ist eine verringerte Arzneimittelabsorption nach bariatrischen Operationen beschrieben. In einer retrospektiven Studie mit 439 Patient:innen, die sich einer Roux-en-Y-Magenbypassoperation unterzogen hatten, stellten Cunningham et al. fest, dass bei 23 % der Patienten eine Erhöhung ihrer Antidepressivadosis postoperativ nötig wurde [3]. Voelker et al. berichten über ein verändertes Verteilungsvolumen aufgrund der Gewichtsabnahme nach einer Magenbypassoperation, was wiederum eine Dosisanpassung der antidepressiven Medikation nötig machte [9]. Roerig et al. führten eine Fall-Kontroll-Pharmakokinetikstudie mit Sertralin durch und fanden in der Gruppe der Personen nach Magenbypassoperation deutlich geringere Wirkstoffspiegel im Vergleich zur nichtoperierten Kontrolle [7]. Insgesamt existieren einige Hinweise auf eine postoperativ bedingte veränderte Antidepressivawirkung wie bei unserem Patienten, allerdings sind weitere klinische Studien nötig, um die zugrunde liegenden Mechanismen zu verstehen.

Der hier vorgestellte Fall zeigt, dass bei einem Nichtansprechen auf eine antidepressive Medikation leitliniengerecht ein TDM durchgeführt werden sollte. Können hierbei keine Medikamentenspiegel nachgewiesen werden, sollte auch an seltene Ursachen wie eine gastrointestinale Resorptionsstörung nach abdominellem operativem Eingriff gedacht und entsprechend anamnestisch erhoben und interdisziplinär diskutiert werden.

## Fazit für die Praxis

- Bei einer TRD sollte zunächst eine ausführliche Ursachenforschung inkl. TDM und Erhebung aller Vorerkrankungen und durchgeführter Operationen erfolgen.
- Bei abdominellen Operationen in der Vorgeschichte sollte auch an eine gastrointestinale Resorptionsstörung gedacht und interdisziplinär diskutiert werden.
- Ein Wechsel der Applikationsform des Antidepressivums kann bei einer gastrointestinal bedingten Resorptionsstörung zu einem Ansprechen auf die Medikation führen.
